# Benzothiazole-Phthalimide Hybrids as Anti-Breast Cancer and Antimicrobial Agents

**DOI:** 10.3390/antibiotics12121651

**Published:** 2023-11-23

**Authors:** Alexia Barbarossa, Jessica Ceramella, Alessia Carocci, Domenico Iacopetta, Antonio Rosato, Francesco Limongelli, Antonio Carrieri, Daniela Bonofiglio, Maria Stefania Sinicropi

**Affiliations:** 1 Department of Pharmacy—Pharmaceutical Sciences, University of Bari “Aldo Moro”, 70125 Bari, Italy; alexia.barbarossa@uniba.it (A.B.); antonio.rosato@uniba.it (A.R.); francesco.limongelli@uniba.it (F.L.); antonio.carrieri@uniba.it (A.C.); 2 Department of Pharmacy, Health and Nutritional Sciences, University of Calabria, 87036 Arcavacata di Rende, Italy; jessica.ceramella@unical.it (J.C.); daniela.bonofiglio@unical.it (D.B.); s.sinicropi@unical.it (M.S.S.)

**Keywords:** anticancers, antimicrobials, benzothiazoles, thalidomide, antioxidant activity, drug repositioning

## Abstract

The benzothiazole nucleus is a major heterocyclic scaffold whose therapeutic potential has been thoroughly explored due to its structural simplicity and ease of synthesis. In fact, several benzothiazole derivatives have been synthesized over time, demonstrating numerous pharmacological properties such as anticancer, antimicrobial, anti-inflammatory, and antioxidant activities. Herein, we propose a new series of benzothiazole-phthalimide hybrids obtained by linking the phthalimide moiety to differently substituted benzothiazole nuclei through the N atom. These compounds have been screened for their anticancer properties against two human breast cancer cell lines. Furthermore, we delved into the mechanism of action of the most active hybrid, compound **3h**, by assessing its capability to damage the nuclear DNA, trigger the apoptotic process in the high metastatic MDA-MB-231 cells, and prevent cellular migration. Moreover, in view of the documented antimicrobial activities of the two scaffolds involved, we explored the antibacterial and antifungal effects of the studied compounds by means of the broth microdilution method. Among the studied compounds, **3h** showed the highest antimicrobial activity, both against gram-positive and gram-negative bacterial strains belonging to the ESKAPE pathogens (*Enterococcus faecium*, *Staphylococcus aureus*, *Klebsiella pneumoniae*, *Acinetobacter baumannii*, *Pseudomonas aeruginosa*, and *Enterobacter* species) and against fungal strains of the *Candida* species with MIC_s_ values ranging from 16 to 32 µg/mL.

## 1. Introduction

**Figure 1:**
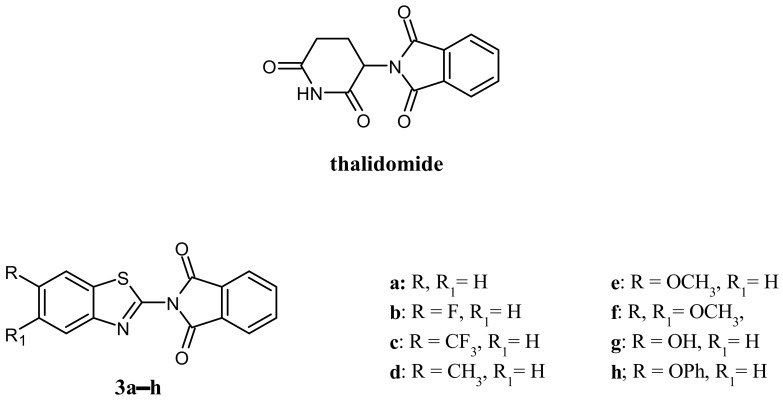
Structure of thalidomide and the compounds under study.

The number of individuals affected by oncological diseases and infections caused by multi-drug-resistant pathogens has dramatically increased in recent years; thus, the discovery of new treatment options and strategies represents a challenge for current research. Nearly seven million people worldwide die from cancer each year, making it an extremely serious global health problem. Due to its high incidence and morbidity, breast cancer is among the most medically significant ones [1]. Even though significant advancements in the diagnosis and treatment of different types of cancer have been made, the cytotoxic effects of most anticancer agents and the occurrence of cellular drug resistance are still the main hurdles to successful chemotherapy [2]. Therefore, the development of new, more selective antineoplastic agents with less toxicity and the discovery of novel biological targets are some of the most urgent needs. In addition, patients with neoplastic disorders are predisposed to microbial infections because of the immunosuppression caused by anticancer drug therapy. Meanwhile, an additional issue of global concern is represented by the spread of certain pathogenic bacteria and opportunistic fungi that have developed resistance to the currently marketed antimicrobial agents [3]. Particularly, the antimicrobial-resistant pathogens defined as ESKAPE (*Enterococcus faecium*, *Staphylococcus aureus*, *Klebsiella pneumoniae*, *Acinetobacter baumannii*, *Pseudomonas aeruginosa*, and *Enterobacter* species) represent one of the major threats both in hospitals and in the community [4]. ESKAPE pathogens have acquired antimicrobial resistance genes, resulting in fewer treatment options for serious infections and a greater disease burden caused by treatment failure. The development of new antimicrobial therapies has been rekindled due to this looming health threat [5]. In such a scenario, the availability of drugs that could be used for patients suffering from cancer and a microbial infection would be ideally suitable from both therapeutic and cost-effective points of view, with the hope of an improved response and few side effects. Heterocyclic compounds represent one of the largest families of organic compounds with a wide range of applications [6]. Indeed, they have been identified as the fundamental building blocks of medicinal chemistry and are present in a variety of medicines with distinctive physicochemical features. The benzothiazole core, due to its structural simplicity and ease of synthesis, is a major heterocyclic scaffold whose therapeutic potential has been thoroughly explored in medicinal chemistry [7,8]. Indeed, benzothiazole derivatives show a broad spectrum of attractive pharmacological properties, including antitumor [9], antimicrobial [10], anti-inflammatory [11], antiviral [12], and antioxidant [13,14] activities, thus representing an important class of biologically active molecules. Another core of great interest in pharmaceutical chemistry is the phthalimide nucleus, used as a privileged structure in drug design; indeed, due to their wide range of chemotherapeutic potential, particularly their antineoplastic activity, compounds bearing a phthalimide core have drawn a lot of attention over time. [15]. This nucleus constitutes the main pharmacophoric feature of thalidomide (Figure 1), an old, widely known drug initially used as an antiemetic in pregnant women. However, this drug was removed from the market in 1962 due to its severe side effects on normal fetal growth [16]. Nevertheless, the academic community has extensively examined the potential for thalidomide repositing in various health areas [17]. Indeed, many studies have demonstrated the immunomodulatory [18,19], anticancer [15,20], anti-inflammatory [21], anti-angiogenic [22], antimicrobial [23], and antiviral activities [24] of thalidomide and its derivatives. Thus, thalidomide appears to be a multi-target drug and a model for the design of novel drugs. Unfortunately, thalidomide administration suffers from several side effects, such as asthenia, rashes, constipation, sleepiness, neuropathy, leucopenia, and hepatotoxicity, in addition to its devastating teratogenic effects [25]. This latter adverse effect is thought to be inherent to the chemical structure of the substance, which is characterized by the presence of a chiral carbon that produces two distinct enantiomers that interconvert in vivo [26]. Several studies indicate that cereblon (CRBN) is the direct target of thalidomide, being implicated in both its beneficial and teratogenic effects. Indeed, CRBN is a substrate receptor of the cullin-4 really interesting new gene (RING) E3 ligase complex, whose mRNA is highly expressed in the cerebellum as well as in many cancerous cells [27]. Structural and biochemical studies determined that the S enantiomer shows a 10-fold higher affinity to CRBN than the R-isomer, indicating the teratogenic effect produced by the S-isomer [28]. In that light, many thalidomide analogues have been developed and investigated; nevertheless, these substances still bear a chiral carbon and are only somewhat useful since they produce central neurotoxicity [29]. On the basis of these considerations, in order to overcome the structural and pharmacological drawbacks of thalidomide, we synthesized a small series of achiral compounds (Figure 1), structurally related to thalidomide, obtained by fusing a phthalimide nucleus to a benzothiazole core. The anticancer activity of these new compounds against two human breast cancer cell lines, MDA-MB-231 and MCF-7 cells, is reported herein. In addition, their ability to damage the nuclear DNA leading to apoptotic cell death and prevent cancer cell migration, along with the antioxidant potency of the lead compound, has been studied. The potential antimicrobial activity of these benzothiazole-phthalimide hybrids against ESKAPE pathogens and *Candida* species strains was also evaluated.

## 2. Results

### 2.1. Chemistry

**Scheme 1:**
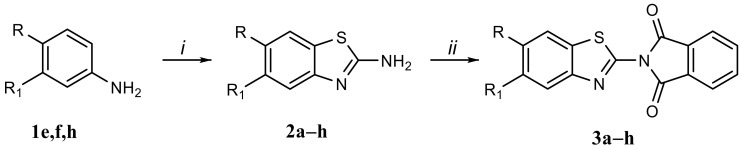
*Reagents and conditions*: (i) Br_2_, NH_4_SCN, glacial CH_3_COOH, 0 °C → r.t., 24 h; (ii) phthalic anhydride, Δ, 4–6 h, for **3a**–**e**; or phthalic anhydride, glacial CH_3_COOH, 130 °C, 7 h, for **3f**–**h**.

The compounds under study (**3a–h**) were synthesized as shown in Scheme 1. Non-commercial 2-amino-1,3-benzothiazoles (**2e,f,h**) were synthesized from the corresponding anilines (**1e,f,h**) by reaction with Br_2_, NH_4_SCN in glacial CH_3_COOH. Next, condensation of the amine group of benzothiazoles with phthalic anhydride was carried out through two different procedures. According to the first procedure, the reagents, in equimolecular amounts, were heated in the absence of solvent to the melting temperature (for **3a–e**). The same reaction was conducted in glacial acetic acid for benzothiazoles with a high melting point (**3f–h**). Physiochemical properties of the synthesized compounds have been reported in Table 1.

### 2.2. Biological Results

#### 2.2.1. Cell Viability Assay

The anticancer activity of the studied compounds (**3a–h**) was explored with regards to two human breast cancer cell lines, namely the estrogen receptor positive (ER+) MCF-7 cells and the triple negative MDA-MB-231 cells (ER-, PR-, and HER-2/Neu-). Cells were continuously exposed to the test substances for 72 h, after which their viability was assessed by means of the MTT assay. Table 2 lists the determined IC_50_ values for the investigated substances and the reference drug, thalidomide. As indicated, thalidomide exhibited only mild antitumor activity in both cell lines used in the assay. The IC_50_ values amounted to 413.0 ± 2.0 μM and 360.0 ± 2.0 μM for the MDA-MB-231 and MCF-7 cell lines, respectively, whereas some of the synthesized compounds evidenced a noteworthy effect. Among them, compound **3h** showed the best anticancer activity, particularly against the MDA-MB-231 cells, with an IC_50_ value amounting to 14.0 ± 0.5 μM. The activity of **3h** towards the MCF-7 cell line is also noteworthy, with an IC_50_ value of 26.2 ± 0.9 μM. Compound **3f** bearing two methoxy substituents on the benzothiazole moiety and the hydroxy analogue **3g** also showed interesting anticancer effects towards MDA-MB-231 cells, albeit to a lesser extent, with IC_50_ values of 49.6 ± 1.0 and 63.3 ± 0.7 μM, respectively. These two compounds showed lower activity against MCF-7 cells. Compounds **3a** and **3b**, mostly towards MDA-MB-231 cells, also showed moderate anticancer activity, whereas compound **3c** did not possess any activity against either of the considered cancer cell lines. Additionally, the same test for all the compounds has been performed on nonmalignant breast epithelial cells (MCF-10A) to ascertain their potential cytotoxicity. Interestingly, almost all compounds, except **3b**, did not interfere with the viability of healthy MCF10-A cells, at least up to the concentration of 500 μM and under the conditions used for these experiments.

Compound **3h** showed an IC_50_ value of 193.9 ± 1.0 µM towards the MCF-10A cell line, but this value is about 13- and 7-fold higher than those recorded for MDA-MB-231 and MCF-7 cancer cells, respectively.

Compound **3h** was found to be the most active against the MDA-MB-231 cells, thus it was chosen for further investigations.

#### 2.2.2. Antibacterial Studies

Compounds **3a–h** were examined in accordance with the Clinical Laboratory Standards Institute (CLSI) recommendations [30], by means of the broth microdilution method, against gram-positive bacteria (*Staphylococcus aureus* 25923, *Staphylococcus aureus* 29213, *Staphylococcus aureus* 43300, *Enterococcus faecalis* 29212) and gram-negative bacteria (*Escherichia coli* 25922, *Klebsiella pneumoniae* 13883, *Pseudomonas aeruginosa* 27853) belonging to the ATCC collection, using levofloxacin as a reference drug. Table 3 lists the results, expressed as MIC (µg/mL). Data revealed that all the compounds, except **3d,** were effective against the bacterial strains considered. Among the tested compounds, the most promising results were obtained with the phenoxy substituted benzothiazole derivative **3h**, particularly against *Staphylococcus aureus* ATCC 25,923 strains with a MIC value of 16 μg/mL. Good activity has also been achieved with this compound against the methicillin-resistant *S. aureus* ATCC 43300 and gram-negative bacteria, with a MIC of 32 μg/mL. Interesting results were also obtained by the dimethoxy benzothiazole derivative **3f** and the hydroxy analogue **3g**, which have been shown to be active against gram-positive bacteria in the range of 32–64 μg/mL and against gram-negative strains at 64 μg/mL. It is worth noting the activity of the methoxy benzothiazole derivative **3e** against *S. aureus* ATCC 43,300 with a MIC value of 32 μg/mL. The lack of activity observed for compound **3d** could be conceivably due to its poor solubility in the culture medium containing the inoculum.

#### 2.2.3. Antifungal Studies

In accordance with CLSI guidelines [31], compounds **3a–h** were tested against a variety of fungal strains (*Candida albicans* 10231, *Candida albicans* 90028, *Candida glabrata* 15126, and *Candida parapsilosis* 22019) belonging to the ATCC collection. Amphotericin B was utilized as a reference drug. The results, expressed as MIC (µg/mL), are listed in Table 4. A similar trend of results, or even better than that obtained against bacteria, was assessed against fungal strains. Indeed, all the benzothiazole-phthalimide hybrids, except compound **3d**, showed mild to significant antifungal activity against all the *Candida* spp. considered. Also, in this case, compound **3h** emerged as the best of the series, with MICs of 16 μg/mL against *Candida glabrata* and *Candida parapsilosis* strains and 32 μg/mL against *Candida albicans* strains. Analogues **3f** and **3g,** along with the unsubstituted benzothiazole derivative **3a** and the fluorine derivative **3b,** showed interesting activity against *C. glabrata* and *C. parapsilosis* with MIC values of 32 μg/mL. The best effect against *Candida albicans* 10231 was achieved with compound **3h**.

#### 2.2.4. Compound **3h** Induces DNA Damage in MDA-MB-231 Cells

**Figure 2:**
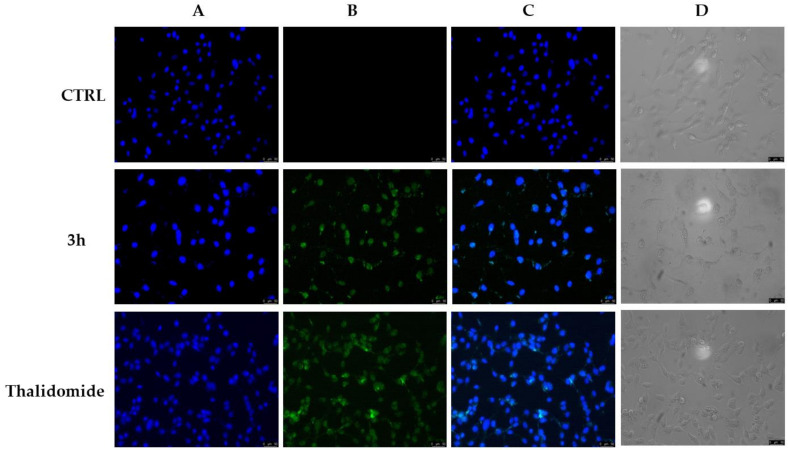
TUNEL assay. MDA-MB-231 cells were exposed to the negative control vehicle (DMSO, CTRL), **3h**, or thalidomide (50 μM concentrations for 24 h), then subjected to the TUNEL assay. The green nuclear fluorescence, visible in **3h-** or thalidomide-treated cells (**B**) and absent in vehicle-treated cells, indicates that DNA damage is occurring. (**A**): nuclear counterstain with DAPI, excitation/emission wavelength 350 nm/460 nm; (**B**): CF™488A excitation/emission wavelength 490 nm/515 nm; (**C**): merge; (**D**): bright field. Images were acquired at 20× magnification, and representative fields of two separate experiments are shown.

To further assess the effect of **3h** treatment in MDA-MB-231, the terminal deoxynucleotidyl transferase dUTP nick-end labeling (TUNEL) assay was used [32]. Thus, **3h** or Thalidomide, as reference drug, were administered to the cells, for 24 h, at 25 and 50 μM concentrations, respectively, or with only the vehicle (DMSO), as negative control and then processed as outlined in the experimental section. Figure 2 depicts the cells treated with a concentration of 50 μM, where **3h** (panel B) promoted DNA damage, as evidenced by the green fluorescence. As expected, thalidomide was found to damage DNA as well, whereas the vehicle-treated cells (CTRL, Figure 2, panel B) did not show any damage. Nuclei were counterstained with blue DAPI (Figure 2, panels A), and merge channels were also shown (Figure 2, panels C), where it is evident the perfect superposition of green and blue signals. These outcomes indicate that **3h** produced DNA fragmentation and suggested that MDA-MB-231 cell death was occurring by the apoptotic mechanism. Images taken from bright fields were also included (Figure 2, panels D).

#### 2.2.5. Effect of Compound **3h** on Cell Migration

**Figure 3:**
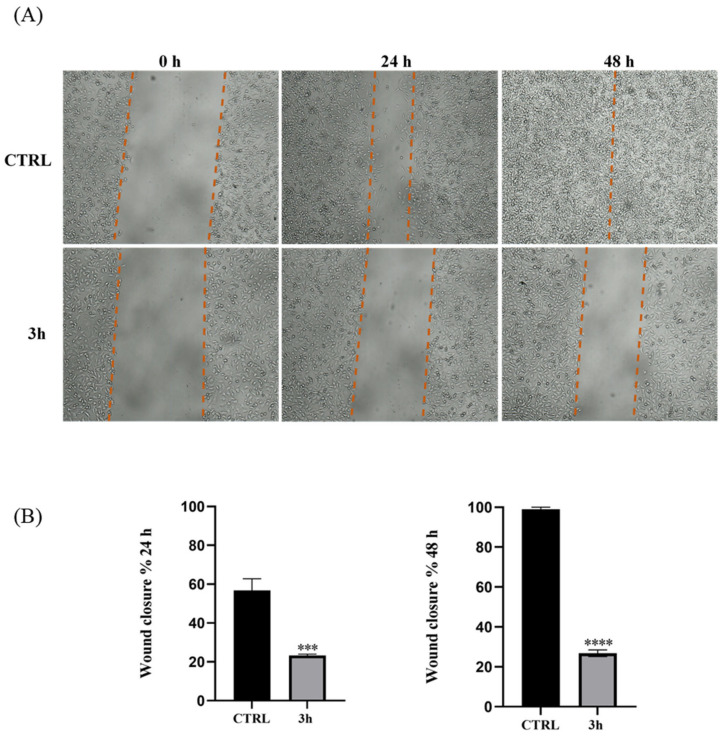
Wound-healing assay conducted on MDA-MB-231 cells treated with compound **3h** or vehicle (Ctrl, DMSO). (**A**): wound closure was monitored at 0, 24, and 48 h using an inverted microscope (4× magnification). Dotted orange lines define the areas that lack cells. (**B**): quantitative analysis of wound closure. The data represent the mean values ± SD of three independent experiments. (*** *p* < 0.001 and **** *p* < 0.0001 vs. CTRL).

Among the compounds under study, compound **3h** was chosen for further investigation since it has been shown to possess higher effectiveness in reducing cell viability, as displayed in the previous experiment. Thus, its ability to prevent the migration of MDA-MB-231 was tested by means of the wound-healing assay. Confluent monolayers of cells were cultivated and plated before being scraped to create a wound (see Experimental Section) and treated with compound **3h** at concentrations corresponding to its IC_50_ value (Table 2) for 24 and 48 h, or DMSO (Ctrl). These distinct endpoint times were experimentally defined since MDA-MB-231 cells have a high growth rate and frequently give rise to metastases in vivo. In fact, 48 h were sufficient for the wound closure in the control cells, treated using only the vehicle. Interestingly, our results suggested that **3h** prevented the total wound closure at percentages of ~23% or ~26% after 24 h and 48 h of treatment, respectively, thus showing a good effect in reducing cancer cell migration (Figure 3).

#### 2.2.6. ROS Scavenging Effect of Compound **3h**

**Figure 4:**
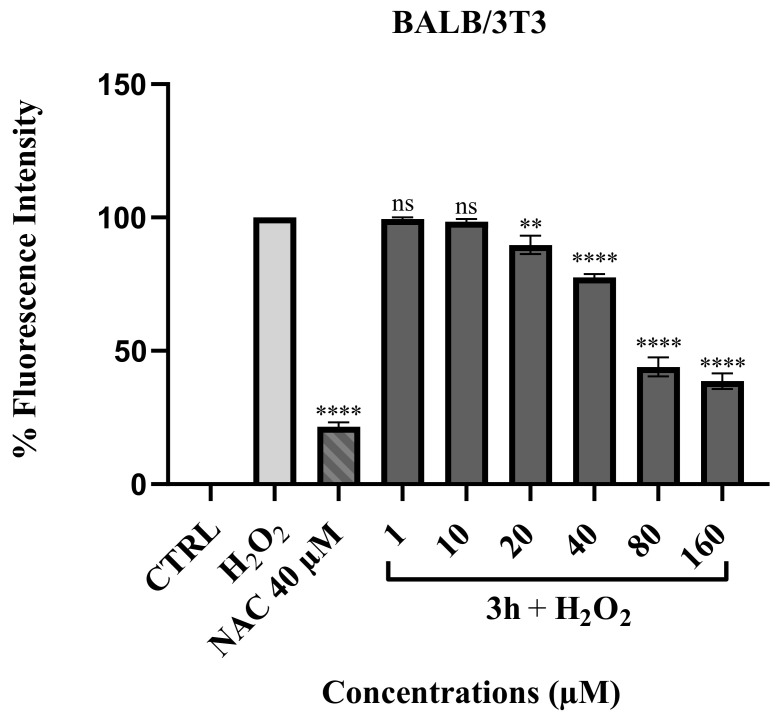
DCFH oxidation in BALB-3T3 cells after 24 h incubation with 40 µM NAC or increasing concentrations (1–160 µM) of compound **3h** in the presence of H_2_O_2_. Bars represent mean values ± SD obtained from three individual experiments performed in triplicate. Significant differences versus H_2_O_2_: not significant differences (ns, *p* > 0.05), ** *p* < 0.01, **** *p* < 0.0001. NAC: *N*-acetylcysteine.

The potential ROS scavenging effect of our most active compound **3h** was explored on a non-tumoral cell model, the mouse fibroblasts BALB/3T3, by means of the 2′,7′-dichlorofluorescin diacetate assay. BALB/3T3 cells were treated with compound **3h** at different concentrations (1–160 μM), for which we have previously assessed the lack of toxicity by means of the MTT assay (IC_50_ > 200 µM). Cells were treated for 24 h, and *N*-acetylcystein (NAC), a compound well-known for its antioxidant capacity, was used as a reference molecule. The findings, shown in Figure 4, revealed that the pre-treatment with compound **3h** markedly decreased the oxidative stress induced by H_2_O_2_ in a dose-dependent manner when compared with the cells treated only with H_2_O_2_. Compound **3h** was able to reduce ROS formation by 61.4 ± 2% at the maximum concentration of 160 μM in the BALB/3T3 cells, as shown by the quantification of fluorescence intensity, proving compound **3h** to be a good ROS scavenger.

## 3. Discussion

The synthesis of new thalidomide analogues that are able to exert multiple effects on cancer cell proliferation and have an improved pharmacological profile compared to the parent compound has been of great interest for medicinal chemistry research in recent years. As part of our ongoing research program [33] directed toward the development of antitumor heterocyclic compounds, among which thalidomide-correlated compounds are included, we attempted to overcome the structural and pharmacological drawbacks of thalidomide by synthesizing a small series of structurally related compounds that lack the chiral carbon of the parent molecule, this being a feature of thalidomide related to its teratogenic effect. These compounds were obtained by directly fusing a phthalimide nucleus to a benzothiazole core through the N atom. In our previous study, two of these hybrids, compound **3a** and **3d** (Figure 1), performed as antiangiogenic agents with reduced side effects compared to thalidomide [18]. In this work, we enlarged the small series of analogues of the previously reported benzothiazole-phthalimide hybrids with the aim of exploring the potential anti-breast cancer effects of these compounds by testing their activity against two human breast cancer cell lines, MDA-MB-231 and MCF-7 cells. The hybrids differ from each other only for the substituents present on the benzothiazole moiety, with this feature deriving from the substituted anilines used as starting materials. Despite the diverse nature of these substituents, it is not possible to clearly relay the activity of the analogues with the different electronic distribution on the aromatic portion of the benzothiazole moiety induced by these substituents. However, compound **3c** bearing a strong electron-withdrawing substituent on the benzothiazole moiety, as the trifluoromethyl one, did not show any activity toward either of the cell lines tested, while its fluorine analogue **3b** showed only moderate anticancer activity. On the other hand, compounds bearing electron-donating substituents on the benzothiazole moiety (**3f** and **3g**) showed an interesting anticancer effect against MDA-MB-231 cells. Among the tested compounds, compound **3h**, bearing the bulky phenoxy group on the benzothiazole moiety, showed the best anticancer activity, mostly against the highly metastatic MDA-MB-231 cells, thus performing as the lead compound of the series. Once it was established that our compounds possess improved antitumor activity with respect to thalidomide, we tested all the considered compounds on nonmalignant breast epithelial cells (MCF-10A) to determine cytotoxic effects, if any. We observed a slight activity towards this cell line only for compounds **3h** and **3b**; however, **3h** shows a good selectivity against the cancer cells adopted with respect to the nonmalignant ones, since its IC_50_ values on the latters are about 13- and 7-fold higher than those recorded for MDA-MB-231 and MCF-7 cancer cells, respectively. The improved capacity of our compounds to affect the proliferation of MCF-7 cells and, most importantly, the highly aggressive and metastatic MDA-MB-231 cell line without significantly affecting non-tumor MCF-10A breast epithelial cells prompted us to investigate in detail the properties of the most active compound. The TUNEL assay, a long-established assay able to detect DNA fragmentation induced by endonucleases during apoptosis [32], was conducted on MCF-7 cells. The results indicated that **3h** produced DNA fragmentation leading to MDA-MB-231 cell death by the apoptotic mechanism. Further investigation on **3h** by means of the wound-healing assay allowed us to assess that this hybrid can prevent the migration of MDA-MB-231 cells, which provides an estimate of the ability of cancer cells to metastasize. Reactive oxygen species (ROS), at low doses, are known to be crucial factors in regulating normal physiological cell processes. However, when the intracellular sources of ROS are increased, severe impairment of proteins, nucleic acids, lipids, membranes, and other organelles occurs, thus triggering cell death pathways such as apoptosis [34]. Oxidative stress and redox signaling have been involved in the genesis of cancer, and ROS can affect the phenotypic behavior of cancer cells and their responsiveness to therapeutic treatments [35]. DNA oxidative damage can undoubtedly stimulate cancer-causing mutations. Increases in basal levels of oxidative signaling that may drive proliferation and encourage additional mutations are linked to the oncogenic transformation of fibroblasts [36]. Therefore, considering the central role of ROS in the onset of cancer, the availability of anticancer compounds endowed with antioxidant properties to explicate chemo-preventive action could enhance the known chemotherapy protocols. In light of these observations and considering the antioxidant properties of benzothiazole derivatives reported in the literature [13,14], we decided to explore the potential ROS-scavenging effect of our most active compound, **3h**, on a non-tumoral cell model, the mouse fibroblasts BALB/3T3. The obtained result showed that compound **3h** acts as a good scavenging agent for ROS since it is able to reduce ROS generation induced by H_2_O_2_ in a dose-dependent manner.

In addition, considering the importance of both phthalimide and benzothiazole nuclei in the antimicrobial field, as widely reported in the literature [37,38], we also evaluated these series of compounds as antibacterial agents on different representative gram-positive and gram-negative bacterial strains, particularly focusing on bacteria classified as ESKAPE pathogens. All the compounds, except **3d,** were effective against the bacterial strains considered. The lack of activity observed for compound **3d** could be conceivably due to its poor solubility in the culture medium containing the inoculum. Once again, compound **3h** performed as the best in the series, particularly against *Staphylococcus aureus* ATCC 25,923 strains with a MIC value of 16 μg/mL. The potential antifungal activity was tested against strains of the *Candida* species, whose infections are of great concern as their incidence has significantly increased in recent years, especially in immunocompromised patients [39]. A similar trend of results, or even better than that obtained against bacteria, was assessed against fungal strains. Indeed, all the benzothiazole-phthalimide hybrids, except compound **3d**, showed mild to significant antifungal activity against all the *Candida* spp. considered. Also, in this case, compound **3h** emerged as the best compound of the series. Taken together, these outcomes suggest compound **3h** as a good candidate as an anticancer and antimicrobial agent. However, further investigation must be carried out to elucidate its mechanisms of action. Indeed, as we demonstrated, it induces DNA damage in MDA-MB-231 cells and MCF-7). Since DNA damage can be triggered by several stimuli interfering with different intracellular targets, it is quite impossible to speculate about or hypothesize only one mechanism for this compound, including the variation of ROS levels. Therefore, multiple pathways could be involved in the observed wide range of activities. We proved that, in cancer cells, exposure to compound **3h** leads to death by apoptosis; however, it is possible that it can also trigger the apoptotic process in microbial cells, which would be the object of further investigation, as well as the involvement of oxidative stress pathways of the bacterial and fungal species in the antimicrobial activity of our lead.

## 4. Materials and Methods

### 4.1. Chemistry

All chemicals were purchased from Sigma-Aldrich (St. Louis, MO, USA) or Lancaster (Lancaster Synthesis Ltd., White Lund Industrial Estate, Lancashire, UK) at the highest quality commercially available. Solvents were reagent-grade unless otherwise indicated. Yields refer to purified products that were not optimized. The structures of the compounds were confirmed by routine spectrometric and spectroscopic analyses. Only spectra for compounds not previously described are given. Melting points were determined on a Gallenkamp melting point apparatus (Weiss-Gallenkamp, London, UK) in open glass capillary tubes and were uncorrected. ^1^H and ^13^C NMR spectra were recorded on a Varian VX Mercury spectrometer (Varian Inc., Palo Alto, CA, USA) operating at 300 and 75 MHz for ^1^H and ^13^C, respectively, or an Agilent 500 MHz operating at 500 and 125 MHz for 1H and 13C, respectively, using CDCl_3_, CD_3_OD, or DMSO-*d6* as solvent. Chemical shifts (δ) are reported in ppm relative to the residual non-deuterated solvent resonance: CDCl_3_, δ = 7.26 (^1^H NMR) and δ = 77.3 (^13^C NMR); CD_3_OD, δ = 3.30 (^1^H NMR) and δ = 47.8 (^13^C NMR); DMSO-*d6*, δ = 2.48 (^1^H NMR) and δ = 39.5 (^13^C NMR) as internal references. Coupling constants (J) are given in Hz. Gas chromatography (GC)/mass spectroscopy (MS) was performed on a Hewlett-Packard 6890–5973 MSD (Hewlett-Packard, Palo Alto, CA) at low resolution. Elemental analyses were performed on a Eurovector Euro EA3000 analyzer, and the data for C, H, and N were within ± 0.4 of theoretical values. Chromatographic separations were performed on silica gel columns by column chromatography on silica gel (Kieselgel 60, 0.040–0.063 mm, Merck, Darmstadt, Germany). TLC analyses were performed on precoated silica gel on aluminum sheets (Kieselgel 60 F254, Merck, Darmstadt, Germany).

2-Amino-6-methoxy-1,3-benzothiazole (**2e**). 6-Methoxyaniline (**1e**) (1.00 g, 8.1 mmol) and NH_4_SCN (2.47 g, 32.5 mmol) were dissolved in glacial acetic acid (15 mL) and cooled in an ice bath with stirring under N_2_. After that, bromine (0.42 mL dissolved in 4 mL of glacial acetic acid) was added dropwise, protecting the reaction mixture from light. The mixture was stirred at room temperature for 24 h. Sodium hydroxide pellets and ice were added with stirring until pH 11 was attained, and the mixture was extracted with EtOAc. The organic layer was dried over anhydrous Na_2_SO_4_, and the solvent was evaporated in vacuo to give 1.404 g of a brown solid (96%) that was recrystallized from CHCl_3_/hexane: mp 169–170 °C; GC-MS (70 eV) m/z (%) 180 (M^+^, 81), 165 (100); ^1^H NMR (500 MHz, DMSO-*d6*): δ 3.60 (s, 3H, OCH_3_), 7.10 (s, 2H, NH_2_), 7.50–7.70 (m, 3H, Ar); ^13^C NMR (126 MHz, CDCl_3_): δ 55.9 (1C), 105.3 (1C), 113.7 (1C), 119.7 (1C), 132.7 (1C), 146.1 (1C), 155.7 (1C), 163.9 (1C).

5,6-Dimethoxy-1,3-benzothiazole (**2f**). It was prepared as reported for **2e,** starting from 3,4-dimethoxyaniline (**1f**). Yield: 74%; mp: 227–229 °C; GC/MS (70 eV) m/z (%): 210 (M^+^, 100); ^1^H NMR (300 MHz, CDCl_3_): δ 3.72 ppm (s, 6H, OCH_3_), 6.98 (s, 1H), 7.19 (s, 2H), 7.28 (s, 1H). Other spectroscopic data agreed with the literature [40].

2-Amino-6-phenoxy-1,3-benzothiazole (**2h**). It was prepared as reported for **2e,** starting from 4-phenoxyaniline (**1h**). Yield: 65%; mp: 171–172 °C (EtOAc/hexane); GC/MS (70 eV) m/z (%): 242 (M^+^, 100); Anal. calcd. for C_13_H_10_N_2_OS.0.20H_2_O (245.90) %: C 63.50; H 4.26; N 11.39. Found: C 63.80; H 3.89; N 11.41.

2-(1,3-benzothiazol-2-yl)-1H-isoindole-1,3(2H)-dione (**3a**). Equimolar amounts of 2-amino-1,3-benzothiazole (**2a**) (1.000 g, 6.7 mmol) and phthalic anhydride (0.980 g, 6.7 mmol) were heated in the absence of solvent to the benzothiazole melting temperature (170 °C) for 3 h. The mixture was occasionally stirred, and phthalic anhydride, which was sublimed, was pushed down into the reaction mixture until the fusion was complete. The mixture was kept undisturbed for 5 min to allow the liquid mass to solidify. The solid mass was suspended in water to remove unreacted anhydride. The residue was washed with a mixture of water and 96% ethanol (in a ratio of 1:1) and filtered. The white solid obtained was recrystallized from chloroform/hexane providing 0.460 g of white crystals (25%): mp 249–250 °C; GC-MS (70 eV) m/z (%) 280 (M^+^, 100); ^1^H NMR (300 MHz, CDCl_3_): δ 7.44 (t, *J* = 7.6 Hz, 1H), 7.54 (t, *J* = 7.3 Hz, 1H), 7.87–7.93 (m, 3H), 8.05–8.08 (m, 2H), 8.15 (d, *J* = 8.3 Hz, 1H); ^13^C NMR (75 MHz, CDCl_3_): δ 121.2 (1C), 123.4 (1C), 124.6 (2C), 125.4 (1C), 126.4 (1C), 131.1 (2C), 132.9 (2C), 135.4 (1C), 149.5 (1C), 151.9 (1C), 164.5 (2C); Anal calcd for C_15_H_8_N_2_O_2_S (280.03): C 64.27; H 2.88; N 9.99. Found: C 63.98; H 2.87; N 9.95.

2-(6-Fluoro-1,3-benzothiazol-2-yl)-1H-isoindole-1,3(2H)-dione (**3b**). It was prepared as reported for **3a,** starting from 2-amino-6-fluoro-1,3-benzothiazole (**2b**). Yield: 32% (yellow crystals): mp 234–235 °C (CHCl_3_/hexane); GC-MS (70 eV) m/z (%) 298 (M^+^, 100); ^1^H NMR (500 MHz, CDCl_3_): δ 6.94 (dt, *J* = 9.0, 2.4 Hz, 1H), 7.27 (dd, *J* = 8.0, 2.6 Hz, 1H), 7.56 (dd, *J* = 5.5, 3.0 Hz, 2H), 7.78–7.71 (m, 3H); ^13^C NMR (126 MHz, CDCl_3_): δ 107.3 (1C), 107.5 (1C), 115.0 (1C), 115.2 (1C), 124.4 (1C), 124.5 (1C), 124.6 (1C), 130.9 (1C), 133.9 (1C), 135.4 (1C), 146.0 (1C), 151.4 (1C), 159.5 (1C), 161.4 (1C), 164.3 (1C); Anal calcd for C_15_H_7_FN_2_O_2_S^.^0.25 H_2_O (302.80): C 59.50; H 2.50; N 9.25. Found: C 59.86; H 2.52; N 9.03.

2-[6-Trifluoromethyl-1,3-benzothiazol-2-yl]-1H-isoindole-1,3(2H)-dione (**3c**). It was prepared as reported for **3a,** starting from 2-amino-6-trifluoromethyl-1,3-benzothiazole (**2c**). Yield: 33% (white crystals): mp > 250 °C (CHCl_3_/hexane); GC-MS (70 eV) m/z (%) 348 (M^+^, 100); ^1^H NMR (500 MHz, CD_3_OD): δ 7.69–7.58 (m, 4H, Ar), 7.82 (d, *J* = 8.5 Hz, 1H),7.99–8.02 (m, 1H, Ar), 8.13–8.15 (m, 1H, Ar); ^13^C NMR (75 MHz, CD_3_OD): δ 77.35 (t, *J* = 31.5 Hz, 1C), 118.9 (1C), 119.0 (1C), 120.8 (1C), 123.1 (1C), 127.7 (1C), 128.8 (1C), 130.2 (1C), 130.6 (1C), 132.2 (1C), 132.4 (1C) 136.0 (1 C), 151.0 (1C), 161.3 (1C), 166.6 (1C), 169.2 (1C); Anal calcd for C_16_H_7_F_3_N_2_O_2_S.0.33 H_2_O (354.30): C 54.24; H 2.18; N 7.91. Found: C 54.67; H 2.21; N 7.63.

2-[6-Methyl-1,3-benzothiazol-2-yl]-1H-isoindole-1,3(2H)-dione (**3d**). It was prepared as reported for **3a,** starting from 2-amino-6-methyl-1,3-benzothiazole (**2d**). Yield: 56% (yellow crystals): mp > 250 °C (CHCl_3_/hexane); GC-MS (70 eV) m/z (%) 294 (M^+^, 100); ^1^H NMR (300 MHz, CDCl_3_): δ 2.50 (s, 3H, CH_3_), 7.32 (dd, *J* = 8.3, 1.5 Hz, 1H, Ar), 7.68 (s, 1H, Ar), 7.83–7.88 (m, 2H, Ar), 8.0 (d overlapping m at 8.0–8.06 ppm, *J* = 8.3 Hz, 1H, Ar), 8.0–8.06 (m overlapping d at 8.0 ppm, 2H, Ar); ^13^C NMR (75 MHz, CDCl_3_): δ 21.6 (1C), 120.8 (1C), 122.9 (1C), 124.5 (2C), 128.0 (1C), 131.1 (1C), 133.1 (1C), 135.3 (2C), 135.6 (2C), 147.5 (1C), 150.9 (1C), 164.6 (2C); Anal calcd for C_16_H_10_N_2_O_2_S.0.33 H_2_O (300.33): C 63.99; H 3.58; N 9.33. Found: C 64.01; H 3.58; N 8.88.

2-[6-Methoxy-1,3-benzothiazol-2-yl]-1H-isoindole-1,3(2H)-dione (**3e**). It was prepared as reported for **3a,** starting from 2-amino-6-methoxy-1,3-benzothiazole (**2e**). Yield: 74% (yellow crystals): mp 227–229 °C (CHCl_3_/hexane); GC-MS (70 eV) m/z (%) 310 (M^+^, 100); ^1^H NMR (500 MHz, CDCl_3_): δ 3.89 (s, 3H, CH_3_), 7.10 (dd, *J* = 9.3, 2.4 Hz, 1H, Ar), 77.34 (d, *J* = 2.4 Hz, 1H, Ar), 7.82–7.88 (m, 2H, Ar), 7.98–8.06 (m, 3H, Ar); ^13^C NMR (125 MHz, CDCl_3_): δ 55.8 (1C), 103.7 (1C), 115.7 (1C), 124.1 (1C), 124.5 (2C), 131.1 (1C), 134.5 (1C), 135.3 (4C), 143.9 (1C), 157.9 (1C), 164.6 (2C); Anal calcd for C_16_H_10_N_2_O_3_S.0.33 H_2_O (316.33): C 60.75; H 3.40; N 8.86. Found: C 60.72; H 3.19; N 8.95.

2-[5,6-Dimethoxy-1,3-benzothiazol-2-yl]-1H-isoindole-1,3(2H)-dione (**3f**). A solution of 2-amino-5,6-dimethoxy-1,3-benzothiazole (**2f**) (0.500 g, 1.5 mmol) and phthalic anhydride (0.223 g, 1.5 mmol) in glacial acetic acid (20 mL) was stirred at 130 °C for 7 h under an Ar atmosphere. The reaction mixture was cooled in an ice bath, and then 30 mL of H_2_O were added. The solid residue was filtered and washed with a mixture of water and 96% ethanol (1:1), then it was recrystallized from chloroform/hexane, providing 0.324 g of yellow crystals (63%): mp > 250 °C; GC-MS (70 eV) m/z (%) 340 (M^+^, 100); ^1^H NMR (500 MHz, CDCl_3_): δ 3.96 ppm (s, 6H, OCH_3_), 7.29 (s, 1H, Ar), 7.62 (s, 1H, Ar), 7.85 (dd, *J* = 5.5, 3.0 Hz, 2H, Ar), 8.02 (dd, *J* = 5.5, 3.1 Hz, 2H, Ar); ^13^C NMR (126 MHz, CDCl_3_): δ 56.1 (1C), 56.3 (1C), 102.0 (1C), 105.2 (1C), 124.5 (2C), 125.0 (2C), 131.1 (2C), 135.2 (2C), 143.8 (1C), 148.7 (1C), 149.5 (1C), 150.1 (1C), 164.6 (2C); Anal calcd for C_17_H_12_N_2_O_4_S.0.5 H_2_O (349.36): 58.45; H 3.75; N 8.02. Found: C 58.30; H 3.68; N 7.90.

2-[6-Hydroxy-1,3-benzothiazol-2-yl]-1H-isoindole-1,3(2H)-dione (**3g**). It was prepared as reported for **3f,** starting from 2-amino-6-methoxy-1,3-benzothiazole (**2g**). Yield: 20% (yellow crystals): mp > 250 °C (CHCl_3_/hexane); GC-MS (70 eV) m/z (%) 296 (M^+^, 100); ^1^H NMR (500 MHz, CDCl_3_): δ 6.99 (dd, *J* = 8.8, 2.4 Hz, 1H), 7.42 (d, *J* = 2.3 Hz, 1H), 7.62–7.67 (m, 1H), 7.96–7.91 (m, 2H), 8.02 (dt, *J* = 6.9, 3.5 Hz, 2H), 9.85 (s, 1H); ^13^C NMR (75 MHz, CD_3_OD): δ 105.9 (1C), 116.1 (1C), 123.3 (1C), 124.4 (2C), 131.0 (1C), 135.4 (4C), 142.6 (1C), 149.3 (1C), 155.5 (1C), 164.8 (2C); Anal calcd for C_17_H_8_N_2_O_3_S.0.14 H_2_O (298.87): 60.28; H 2.79; N 9.37. Found: C 60.67; H 3.27; N 8.92.

2-[6-Phenoxy-1,3-benzothiazol-2-yl]-1H-isoindole-1,3(2H)-dione (**3h**). It was prepared as reported for **3f,** starting from 2-amino-6-phenoxy-1,3-benzothiazole (**2h**). Yield: 15% (yellow crystals): mp > 250 °C (CHCl_3_/hexane); GC-MS (70 eV) m/z (%) 372 (M^+^, 100); ^1^H NMR (500 MHz, CDCl_3_) δ 7.08–7.03 (m, 2H), 7.17–7.12 (m, 1H), 7.22 (dd, *J* = 8.9, 2.5 Hz, 1H), 7.41–7.33 (m, 2H), 7.46 (t, *J* = 4.6 Hz, 1H), 7.90–7.83 (m, 2H), 8.10–7.99 (m, 3H); ^13^C NMR (126 MHz, CDCl_3_) δ 110.2 (1C), 118.8 (1C), 119.1 (2C), 123.7 (1C), 124.3 (1C), 124.6 (2C), 129.9 (2C), 131.1 (1C), 134.3 (1C), 135.3 (2C), 145.6 (1C), 150.7 (1C), 155.3 (2C), 157.1 (1C), 164.4 (2C); Anal calcd for C_21_H_12_N_2_O_3_S.0.25 H_2_O (376.90): C 66.92; H 3.34; N 7.43. Found: C 67.11; H 3.33; N 7.69.

### 4.2. Biology

#### 4.2.1. Cell Culture

The four cell lines used in this work (MCF-7, MDA-MB-231, MCF-10A, and 3T3-L1) were obtained from the American Type Culture Collection (ATCC, Manassas, VA, USA). All cell lines were maintained at 37 °C in a humidified atmosphere of 95% air and 5% CO_2_ and periodically screened for contamination. MCF-7, human breast cancer cells estrogen receptor (ER)-positive, and human breast cancer triple negative MDA-MB231 were grown in DMEM-F12 medium containing 2 mmol/L L-glutamine, 1 mg/mL penicillin–streptomycin, and 5% newborn calf serum (NCS) or 5% fetal bovine serum (FBS), respectively. MCF-10A human mammary epithelial cells were cultured in DMEM/F12 medium, supplemented with 5% horse serum (HS) (Eurobio, Les Ullis, Cedex, France), 100 U/mL penicillin/streptomycin, 0.5 mg/mL hydrocortisone, 20 ng/mL hEGF (human epidermal growth factor), 10 mg/mL insulin, and 0.1 mg/mL cholera enterotoxin (Sigma-Aldrich, Milano, Italy). BALB/3T3 murine fibroblast cells were cultured in DMEM high glucose supplemented with 10% bovine calf serum (BCS), 1% L-glutamine, and 100 U/mL penicillin/streptomycin.

#### 4.2.2. Cell Viability

Cell viability was evaluated by means of the 3-(4,5-dimethylthiazol-2-y1)-2,5-diphenyltetra zolium bromide (MTT, Sigma-Aldrich, Milan, Italy) assay [41]. On 48-well plates, cells were seeded, and they were cultured in complete medium. Before being treated, cells were starved in serum-free medium for 24 h to allow cell cycle synchronization. Subsequently, cells were exposed to increasing doses of each compound for 72 h in phenol-red-free media supplemented with 1% serum before fresh MTT was added to each well (final concentration: 0.5 mg/mL). Cells were lysed with DMSO after 2 h of incubation at 37 °C, and optical density at 570 nm was then determined with a microplate reader. Using GraphPad Prism 9 software (GraphPad Inc., San Diego, CA, USA), the mean absorbance for each sample was expressed as a percentage over the control and plotted vs. drug concentrations to establish the IC_50_ values. The given standard deviations (SDs) are indicative of three separate tests carried out in triplicate.

#### 4.2.3. Antibacterial Studies

Following the recommendations of the CLSI (Clinical and Laboratory Standards Institute, 2012), the broth microdilution method was used to determine the in vitro minimum inhibitory concentrations (MICs, µg/mL). [30]. The concentration of the investigated compounds was set to its highest value to create stock solutions. Then, the stock solutions were diluted 1:10 with Cation-Adjusted Mueller Hinton Broth (Oxoid, Milan, Italy). After that, a series of concentrations from 512 g/mL to 2 g/mL were obtained in the wells by performing two-fold serial dilutions in the appropriate test medium. The diluent used to create the stock solution was 100% DMSO. We used the following bacterial strains from the ATCC collection that were available as freeze-dried discs: gram-positive strains such as *S. aureus* ATCC 25923, 29213, and 43300, and *E. faecalis* ATCC 29212, and gram-negative ones such as *E. coli* ATCC 25922, *K. pneuomoniae* ATCC 13883, and *P. aeruginosa* ATCC 27853. To preserve the purity of cultures and to allow their reproducibility, cryovials of all microbial strains were set up in the medium and stored at −80 °C. Mueller Hinton Broth (MHB) was used to prepare pre-cultures of each bacterial strain, which were then incubated for 3–5 h at 37 °C. In accordance with CLSI procedure M7-A9, the turbidity of the bacterial cell suspension was calibrated to 0.5 McFarland Standard using a spectrophotometric approach (OD_625_ nm 0.08–0.10). The standardized suspension was then further diluted (1:100) with MHB to reach 1–2 × 10^6^ CFU/mL. A 200 µL aliquot of the final inoculum was inserted into each well. As a control for growth, several wells containing only inoculated broth were prepared. The plates were incubated at 37 °C for 24 h, and the MIC values were calculated as the lowest concentration of compounds at which there was no optically detectable bacteria growth. Each experiment was carried out in duplicate and repeated twice to determine the MICs. Levofloxacin served as the study’s standard reference antibiotic. Additionally, the antibacterial activity of DMSO alone (used as a diluent for the investigated compounds) was examined; therefore, volumes of this solvent with a concentration of less than 5% were utilized throughout the experimentation.

#### 4.2.4. Antifungal Studies

A microdilution technique described by the Clinical and Laboratory Standards Institute (CLSI, M27-A3) [31] was used to assess the antifungal activity. The following fungal strains, belonging to the ATCC collection and available as freeze-dried discs, were used: *C. albicans* ATCC 10231, *C. albicans* ATCC 90028, *C. glabrata* ATCC 15126, and *C. parapsilosis* ATCC 22019. Stock solutions were prepared and preserved with purity as previously described for the antibacterial studies. Each yeast strain’s pre-cultures were made in Sabouraud broth (SAB) and allowed to grow until they stopped at 37 °C. By using a spectrophotometric technique (530 nm, range 0.12–0.15), the turbidity of the yeast stock suspension was calibrated to 0.5 McFarland Standard. Next, the standardized suspension was diluted first at 1:50 with SAB and then at 1:20 in the same medium to have 1–5 × 10^6^ CFU/mL. A total of 100 µL of inoculum was seeded into each well. As a control for growth, several wells containing only inoculated broth were prepared. The plates were incubated for 48 h at 37 °C, and the MIC values were calculated as the final well with no fungal growth. The plates were placed in an incubator set at a temperature of 37 °C for a duration of 48 h. MIC values were observed based on the absence of fungal growth in the final well. The MICs were calculated by conducting the antifungal test three times in duplicate. As a control, amphotericin B was used.

#### 4.2.5. TUNEL Assay

DNA damage and cell death were analyzed by means of the TUNEL assay as indicated by the manufacturer’s protocols (CF™488A TUNEL Assay Apoptosis Detection Kit, Biotium, Hayward, CA, USA), with few changes, as in [42]. DAPI staining (0.2 μg/mL, Sigma Aldrich, Milan, Italy) was adopted for cell nuclei. Observations were made at 20× magnification using a fluorescence microscope (Leica DM 6000, Leica Microsystem Srl, Buccinasco, Italy). LAS-X software (Version: 3.5.7.23225; Leica Microsystem Srl, Buccinasco, Italy) was employed for the acquisition and processing of the images. Images are representative of two separate experiments from which different fields were acquired.

#### 4.2.6. Wound-Healing Assay

Confluent monolayers of MDA-MB-231 cells formed after the cells were plated on twelve-well plates and grown in full media. Using a standard 200 µL sterile pipette tip, they were injured in a line, and after being washed with phosphate-buffered saline (PBS) for removing cell debris, **3h** was added at its IC_50_ value. Images were taken at time zero (t = 0 h) to capture the initial area of the wound, and at 24 and 48 h (t = h), estimates were made regarding the recovery of the injured monolayer due to cell migration toward the scratched area. A cell imager microscope (JuLi Smart fluorescence, NanoEnTek Inc., Seoul, Republic of Korea) was used to take the pictures. The percentage of wound closure used to express the cell migration toward the wounds was:

$$wound\;closure\left[\%\right]=[({At}_{0}-{At}_{∆h})÷{\left({At}_{0}\right)}^{-1}]\times 100\%$$

for which At_0_ is the area of wound measured immediately after scratching and At_∆h_ is the area of wound measured 48 h after scratching. Vehicle-treated cells were used as a control. The collected images were analyzed with the JuLI^TM^ smart fluorescent cell viewer (NanoEnTek Inc., Seoul, Republic of Korea).

#### 4.2.7. ROS Protection Assay

ROS production was detected using the oxidation-sensitive fluorescent probe 2′,7′-dichlorodihydrofluorescein diacetate (DCFH-DA, D6665; Sigma-Aldrich, St. Louis, MO, USA) by modifying the method described by Wang and James [43,44]. A black 96-well cell culture plate (Costar, Sigma Aldrich, St. Louis, MO, USA) was used to seed viable cells. After 24 h, the cells were treated with different concentrations of compound **3h** (0.1–160 µM) for 1 h at 37 °C in 5% CO_2_. At the end of the treatment, cells were incubated with 2′,7′-dichlorofluorescein diacetate (DCFH-DA) for 30′, used at a concentration of 25 µM, in 5% CO_2_. Subsequently, ROS production was induced by treatment with hydrogen peroxide (H_2_O_2_) in medium without serum for 30′ at 50 μM. DCFH-DA was hydrolyzed by cellular esterases to 2′,7′-dichlorodihydrofluorescein and then oxidized to the green, fluorescent 2′,7′-dichlorofluorescein (DCF) by intracellular H_2_O_2_. The formation of fluorescent dichlorofluorescein (DCF), due to the oxidation of DCFH in the presence of ROS, was read at 530 nm using a microplate reader, the Tecan Infinite M1000 Pro (Tecan, Cernusco S.N., Italy), and DMSO medium was used for control cells. The data were analyzed for statistical significance (*p* < 0.0001) using a one-way ANOVA, followed by Dunnett’s test performed by GraphPad Prism 9. Standard deviations (SDs) are shown. The findings are presented as the mean ± SD of at least three distinct measurements performed in triplicate.

## 5. Conclusions

Drug discovery and development is an expensive process from different points of view, and more recently, drug repositioning has helped to save time and investments in the medicinal chemistry field. Additionally, the possibility of developing highly effective conjugate chemistry approaches represents an attractive strategy for obtaining hybrid compounds that possess novel and multiple biological activities. Taken together, these approaches were demonstrated to be successful in facing the most common issues in several communicable and non-communicable diseases, such as the onset of resistance phenomena and severe side effects. In this study, we reported the synthesis, characterization, and biological evaluation of a series of achiral benzothiazole-phthalimide hybrids structurally related to thalidomide. The investigation of their biological properties revealed good anticancer, antioxidant, and antimicrobial activities, mostly for the identified lead hybrid, which represents a promising scaffold for further developments. Particularly, this lead showed improved anticancer activity with respect to the parent compound, thalidomide, mostly toward the MDA-MB-231 cells, inducing DNA damage and causing cancer cell death. Moreover, it also displayed interesting activity in reducing cancer cell migration. These features were accompanied by important antibacterial and antifungal properties, as well as good antioxidant activity that protected the murine fibroblasts from the experimentally induced oxidative stress. This work reported a preliminary in vitro screening of the designed compounds; thus, further investigations aimed at clarifying the mechanisms of action of the lead compound of the series may be envisaged. Overall, the disclosed biological properties suggest a high potential for the development of drugs able to tackle different aspects of a disease, such as cancer, which is often coupled with opportunistic infections.

## Figures and Tables

**Table 1:** Physiochemical properties of the compounds under study (**3a–h**).

Compounds	MW ^a^	MP ^b^ (°C)	LogS ^c^	LogP ^d^
**3a**	280.30	249–250	–4.81	3.04
**3b**	298.29	234–235	–5.09	3.18
**3c**	348.30	> 250	–5.82	3.93
**3d**	294.33	> 250	–5.32	3.51
**3e**	310.33	227–229	–4.82	2.79
**3f**	340.35	> 250	–4.78	2.54
**3g**	296.30	> 250	–4.39	2.76
**3h**	372.40	> 250	–6.67	4.47

^a^ MW: molecular weight; ^b^ MP: melting point; ^c^ LogS: ChemAxon calculated Log of aqueous solubility; ^d^ LogP: ChemAxon calculated Log of the o/w partition coefficient. Calculator plugins were used for LogS and LogP property prediction and calculation, Marvin 23.14, 2023, ChemAxon, Budapest, Hungary (http://www.chemaxon.com, accessed on 10 November 2023).

**Table 2:** IC_50_ values of compounds **3a–h** and thalidomide expressed in µM ^a^.

Compounds	MDA-MB-231	MCF-7	MCF10-A
**3a**	81.8 ± 0.8	165.5 ± 1.1	>500
**3b**	71.2 ± 1.0	91.5 ± 0.6	151.9 ± 1.1
**3c**	>500	>500	>500
**3d**	202.6 ± 0.6	66.0 ± 1.2	>500
**3e**	102.8 ± 0.9	223.1 ± 0.9	>500
**3f**	49.6 ± 1.0	93.0 ± 1.4	>500
**3g**	63.3 ± 0.7	71.2 ± 0.7	>500
**3h**	14.0 ± 0.5	26.2 ± 0.9	193.9 ± 1.0
**thalidomide**	413.0 ± 2.0	360.0 ± 2.0	>500

^a^ Values are the mean ± SD of three independent experiments performed in triplicate.

**Table 3:** Antibacterial activity of compounds **3a–h** (MIC, µg/mL).

	Microorganisms							
	Gram-Positive				Gram-Negative			
	*S.a.* 25923	*S.a.* 29213	*S.a.* 43300	*E.f.* 29212	*A.b.* 19606	*E.c*. 25922	*K.p.* 13883	*P.a.* 27853
**3a**	64	64	64	64	64	64	128	64
**3b**	64	64	64	64	128	64	128	64
**3c**	128	128	64	64	128	128	128	128
**3d**	>512	>512	>512	>512	>512	>512	>512	>512
**3e**	32	64	32	64	64	64	64	64
**3f**	32	64	32	32	64	32	64	64
**3g**	32	64	32	32	64	64	64	64
**3h**	16	32	32	32	32	32	32	32
**levofloxacin**	0.5	0.5	0.5	2	8	0.06	8	4

Abbreviations: *S.a.*: *Staphylococcus aureus*; *E.f.*: *Enterococcus faecalis*; *A.b.*: *Acinetobacter baumanii*; *E.c.*: *Escherichia coli*; *K.p.*: *Klebsiella pneumoniae*; *P.a.*: *Pseudomonas aeruginosa*.

**Table 4:** Antifungal activity of compounds **3a–h** (MIC, µg/mL).

	Microorganisms			
	*C.a.* 10231	*C.a.* 90028	*C.g.* 15126	*C.p.* 22019
**3a**	64	64	32	32
**3b**	64	64	32	32
**3c**	64	64	64	64
**3d**	>512	>512	>512	>512
**3e**	64	64	64	32
**3f**	32	64	32	32
**3g**	32	64	32	32
**3h**	32	32	16	16
**amph B**	2	1	2	2

Abbreviations: *C.a.*: *Candida albicans*; *C.g.*: *Candida glabrata*; *C.p.*: *Candida parapsilosis*; amph B: amphotericin B.

## Data Availability

Data are contained within the article.
